# Mechanistic Insights into Starch-Polyphenol Complexation: Role of Structural Differences in Galloyl-Based Polyphenols

**DOI:** 10.3390/antiox15060748

**Published:** 2026-06-13

**Authors:** Liang Wang, Leyi Li, Seda Arioglu-Tuncil, Ting He, Kai Wang

**Affiliations:** 1 Guangdong Provincial Key Laboratory of Food Quality and Safety, College of Food Science, South China Agricultural University, Guangzhou 510642, China; 2 Guangzhou Dublin International College of Life Sciences and Technology, South China Agricultural University, Guangzhou 510642, China; 3 Department of Nutrition and Dietetics, Nezahat Keleşoğlu Health Sciences Faculty, Necmettin Erbakan University, 42090 Konya, Türkiye; 4 School of Food & Pharmaceutical Engineering, Zhaoqing University, Zhaoqing 526061, China

**Keywords:** high-amylose maize starch, complex structure, galloyl moieties, non-covalent interactions, agro-processing by-products

## Abstract

Fruit and vegetable processing by-products, such as peels and pomace, are rich in antioxidant polyphenols and represent promising sources of functional ingredients, but how their galloyl-based polyphenols interact with starch remains insufficiently understood. In this study, corilagin with three non-free galloyl moieties and 1,2,3,4,6-O-pentagalloyl glucose with five free galloyl moieties were used as model polyphenols to clarify how galloyl moiety number and accessibility modulate their complexation with high-amylose maize starch (HAMS). Size-exclusion chromatography showed that both polyphenols preferentially complexed with amylose, while FTIR confirmed that complex formation occurred mainly through non-covalent interactions. The two polyphenols induced distinct changes in HAMS structure. Corilagin disrupted short-range order and produced no detectable crystalline structure, whereas 1,2,3,4,6-O-pentagalloyl glucose enhanced molecular order and induced V-type crystallization. Isothermal titration calorimetry revealed more binding sites but weaker affinity for corilagin, with thermodynamic signatures indicating hydrogen bonding and van der Waals interactions. By contrast, 1,2,3,4,6-O-pentagalloyl glucose showed stronger affinity and hydrophobic interaction-dominated binding. Molecular dynamics simulations further confirmed that 1,2,3,4,6-O-pentagalloyl glucose formed a more stable association with the amylose helix than corilagin. These results indicate that galloyl moiety characteristics markedly influence starch–polyphenol interaction mechanisms, providing guidance for the utilization of polyphenol-rich agro-processing by-products in functional starch-based foods.

## 1. Introduction

Fruit and vegetable processing by-products, such as peels, pomace, seeds, and press residues, are generated in large quantities during food processing and are increasingly recognized as valuable sources of antioxidant polyphenols. These by-products contain diverse phenolic compounds that may contribute to antioxidant activity and other health-related functions, offering potential for their reutilization as functional food ingredients. However, the effective incorporation of by-product-derived polyphenols into food matrices requires a better understanding of their interactions with major food macromolecules. Among these macromolecules, starch is one of the important dietary carbohydrates and is widely used in food and industrial applications [1]. However, native starch has inherent limitations, including high digestibility, low thermal stability, and undesirable textural and pasting properties, which restrict its broader application [2,3]. To overcome these limitations, increasing attention has been paid to the interactions between starch and food components, including lipids, proteins, polysaccharides, and polyphenols, during food processing [4,5,6]. Such interactions can alter starch chain organization, ordered structure, and digestive behavior, and therefore provide an effective approach for modulating the physicochemical and nutritional properties of starch-based foods.

Polyphenols are natural small-molecule compounds widely present in plant-based foods and many agro-processing by-products, including fruits, vegetables, grains, peels, and pomace [7]. Because of their aromatic rings and multiple hydroxyl groups, polyphenols can interact with starch through non-covalent forces, including hydrogen bonding, van der Waals interactions, and hydrophobic interactions [1,2,8,9,10]. These interactions may affect starch chain rearrangement, ordered structure formation, and crystalline organization, thereby leading to different structural and functional responses in starch-based systems [1,11,12]. However, the effects of different polyphenols on starch are not always consistent, suggesting that variations in polyphenol structure may result in distinct interaction patterns with starch [13,14].

Galloyl-based polyphenols represent an important class of polyphenols, including compounds such as gallic acid and tannic acid, all of which contain one or more galloyl moieties in the molecular structure [15,16]. Significant differences exist in the number and linkage pattern of galloyl moieties among different galloyl-based polyphenols [17]. For example, when one galloyl moiety is covalently linked to another galloyl moiety through bonds such as C–C linkages, it is referred to as an unfree galloyl moiety. Conversely, when a galloyl moiety is not directly linked to another galloyl unit, it is referred to as a free galloyl moiety. Such structural differences may further alter molecular flexibility and hydroxyl group accessibility, thereby affecting the interaction behavior and biological activities of galloyl-based polyphenols [18,19]. Previous studies have mainly examined the interactions between starch and individual phenolic compounds, while the role of specific galloyl structural features in starch–polyphenol complexation remains insufficiently understood. In particular, the effects of galloyl number and galloyl accessibility on the binding behavior of galloyl-based polyphenols with starch, and on the subsequent reorganization of starch structure, have not been fully clarified. This knowledge gap limits the rational design of starch–polyphenol complexes with controllable structural and functional properties. Since galloyl groups provide multiple phenolic hydroxyl sites for hydrogen bonding and other non-covalent interactions with starch chains, especially amylose, differences in galloyl configuration may lead to distinct complexation modes and structural outcomes. These structural changes may further affect the short-range order, crystalline structure, and functional performance of starch-based systems.

In this study, corilagin, containing three non-free galloyl moieties, and 1,2,3,4,6-O-pentagalloyl glucose, containing five free galloyl moieties, were selected as model compounds to form complexes with high-amylose maize starch. These two galloyl-based polyphenols possess well-defined structural differences in galloyl number and accessibility, while both contain multiple phenolic hydroxyl groups capable of participating in hydrogen bonding and other non-covalent interactions with starch chains. Therefore, they provide suitable model systems for investigating how galloyl structural features affect starch–polyphenol complexation and the associated structure–function relationships. The molecular structure of complexes was studied using size-exclusion chromatography (SEC). The short-range ordered structure was characterized by Fourier transform infrared spectroscopy (FTIR), and the crystalline structure was analyzed by X-ray diffraction (XRD). The interaction type and binding force between starch and polyphenols were investigated by isothermal titration calorimetry (ITC). Additionally, molecular dynamics (MD) simulation was performed to provide theoretical and visualized insights into the interaction process at the molecular level. The outcomes would provide insights into how the structure of galloyl-based polyphenols affects their interactions with starch, and facilitate the development of high-quality starch–polyphenol-based functional foods.

## 2. Materials and Methods

### 2.1. Materials

High-amylose maize starch (HAMS, amylose content ~61%) was purchased from Shandong Huanong Special Corn Development Co., Ltd. (Dezhou, China). Corilagin (3G′) and 1,2,3,4,6-O-pentagalloyl glucose (5G) were purchased from Yuanzhi Biotech Co., Ltd. (Nanjing, China). Isoamylase (from *Pseudomonas* sp., 200 U/mL) was purchased from Megazyme Ltd. (E-ISAMY, Wicklow, Ireland). All other chemicals were of analytical reagent grade and used as received.

### 2.2. Preparation of Starch–Polyphenol Complexes

The HAMS-3G’ and HAMS-5G complexes were prepared separately according to a method reported previously [20]. High-amylose maize starch (1.0 g) was dispersed in 20 mL of 95% (*v*/*v*) aqueous dimethyl sulfoxide (DMSO) and heated in a boiling water bath with constant stirring for 1 h. Corilagin (3G′, 100 mg) or 1,2,3,4,6-O-pentagalloyl glucose (5G, 100 mg), each dissolved separately in 1 mL of 1% (*v*/*v*) DMSO, was then added to the starch paste and stirred at 90 °C for 15 min to prepare the corresponding complex. Subsequently, deionized water (90 °C, 15 mL) was added, and the mixture was further incubated at 90 °C for 15 min. After storage at room temperature for 24 h, the resulting precipitate was collected by centrifugation, washed three times with 50% (*v*/*v*) ethanol, rinsed with absolute ethanol, dried in an oven at 40 °C, ground into fine powder, and stored for further analysis.

### 2.3. Size-Exclusion Chromatography

Size-exclusion chromatography (SEC) was employed to determine the chain-length distribution of the complexes using an Agilent 1260 Infinity system (Agilent, Santa Clara, CA, USA), based on a previously reported procedure [21]. The samples were debranched with isoamylase prior to analysis and then separated through a series of GRAM columns, including GRAM Pre, GRAM 100, and GRAM 1000 columns (PSS, Mainz, Germany). Calibration was performed using pullulan standards with molecular weights ranging from 342 to 2.35 × 10^6^, allowing the elution volume to be converted into the hydrodynamic radius (Rh) of starch molecules. The degree of polymerization (DP, termed as X) of the debranched chains was then calculated from Rh using the Mark–Houwink equation, as previously described [22]. The resulting chain-length distributions were expressed as SEC weight distributions, w(logX), as a function of X. The amylose content of starch complexes was determined by calculating the proportion of the amylose fraction area relative to the total area under the chain-length distribution curve.

### 2.4. Fourier Transform Infrared Spectroscopy

Fourier transform infrared (FTIR) spectra of the HAMS-3G′and HAMS-5G complexes were acquired using an FTIR spectrometer (Vertex 70, Bruker, Germany) equipped with an RT-DLaTGS detector over the wavenumber range of 4000~400 cm^−1^. A total of 64 scans was collected for each sample at a spectral resolution of 4 cm^−1^. Deconvolution of the 1200~800 cm^−1^ region was performed using OMNIC 9.2 software (Thermo Electric Corporation, Chicago, IL, USA). The calibrated peak heights, used for short-range ordered structure, at 1047 and 1022 cm^−1^ were calculated by the software. The ratio of absorbance at 1047 to 1022 cm^−1^ (labeled as R_1047/1022_) was applied to characterize the short-range ordered structure of the starch complexes [23].

### 2.5. Wide-Angle X-Ray Diffractometry

Wide-angle X-ray diffractometry (XRD) patterns of the HAMS-3G’ and HAMS-5G complexes were recorded using an X-ray diffractometer (D8-Advance, Bruker, Germany) at 40 kV and 40 mV. Scanning was recorded within a 2θ range of 4°~35° with a step size of 0.02° and a scanning rate of 10°/min. The relative crystallinity of the starch complexes was quantified as the proportion of crystalline peak area to the total diffraction area using MDI JADE software (version 6.0, Materials Data Inc., Livermore, CA, USA) [16].

### 2.6. Isothermal Titration Microcalorimetry

The thermodynamic parameters for the interactions of high-amylose maize starch with corilagin and 1,2,3,4,6-O-pentagalloyl glucose were determined at 323.15 K using a MicroCal ITC 200 calorimeter (Malvern Instruments Ltd., Malvern, UK) with MicroCal ORIGIN 7 software, following a previously reported method [24]. High-amylose maize starch (100 mg) was dispersed in 3.75 mL of 10% (*v*/*v*) DMSO solution and heated at 121 °C for 30 min. Corilagin and 1,2,3,4,6-O-pentagalloyl glucose were separately dissolved in the same solvent at a concentration of 0.1 mg/mL. The high-amylose maize starch solution was placed in the sample cell, and the polyphenol solution was loaded into the injection syringe. A total of 19 injections were carried out, with an initial injection volume of 0.4 μL followed by 18 successive injections of 2 μL each. The interval between injections was 60 s, and the stirring speed was maintained at 1000 rpm throughout the titration. Control experiments were performed by injecting the corresponding polyphenol solution into 10% (*v*/*v*) DMSO to correct for the heat of dilution.

### 2.7. Molecular Dynamics Simulation

Molecular dynamics (MD) simulations of the starch–polyphenol systems were performed using the GROMACS 2020.4 [25] with the CHARMM36-feb2021 force field [26]. An amylose model consisting of 26 glucose residues in a left-handed helical conformation was employed as a representative structure of starch to investigate interactions between starch and polyphenols. The amylose model was obtained from the Shanghai Institute of Technology (Shanghai, China). The chemical structures of corilagin and 1,2,3,4,6-O-pentagalloyl glucose were retrieved from the PubChem database (https://pubchem.ncbi.nlm.nih.gov/, accessed on 10 October 2023). The amylose and polyphenol molecules were solvated in a cubic box (11.5 × 11.5 × 11.5 nm^3^) using the TIP3P water model, with a minimum distance of 1.2 nm between the solute and the box edge. The equations of motion were integrated using the leap-frog algorithm with a time step of 2 fs. Each system was simulated for 100 ns (50 million steps), and 10,000 frames were collected for analysis. All bonds involving hydrogen atoms were constrained using the LINCS algorithm (lincs_iter = 1, lincs_order = 4). System temperature was maintained at 363 K using the V-rescale thermostat, and system pressure was controlled at 1 bar using the Parrinello–Rahman barostat with isotropic coupling and a time constant of 2 ps. The non-bonded interactions, including Lennard-Jones and electrostatic interactions, were calculated with a cutoff distance of 0.9 nm. Hydrogen bonds were identified using a donor–acceptor distance cutoff of 0.35 nm and a hydrogen-donor–acceptor angle cutoff of 30° [27].

### 2.8. Statistical Analysis

Data are expressed as mean ± standard deviation from at least triplicated measurements. Statistical analysis was conducted using one-way ANOVA in SPSS software (version 16.0, SPSS Inc., Chicago, IL, USA). Significant differences among mean values were considered at *p* < 0.05.

## 3. Results

### 3.1. Chain-Length Distribition

**Figure 1:**
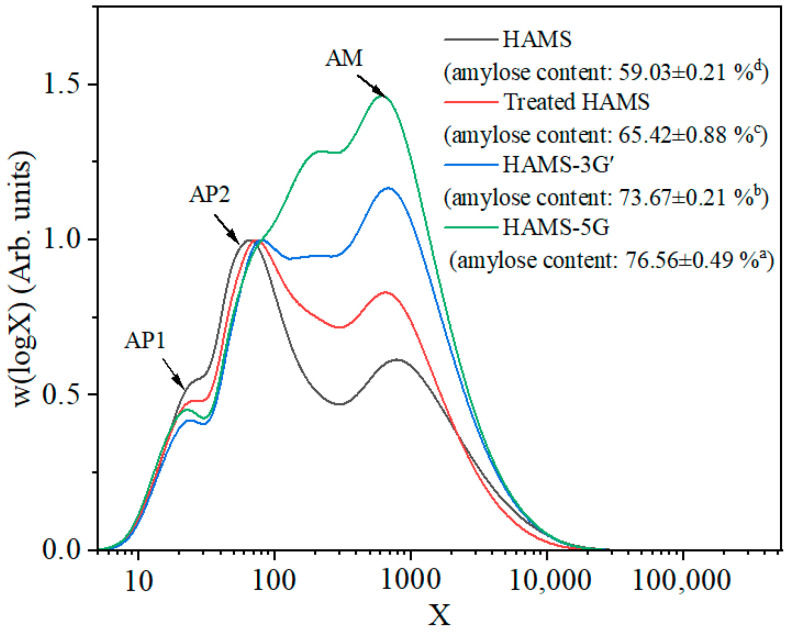
SEC chain-length distributions and amylose content of debranched high-amylose maize starch (HAMS), treated HAMS, HAMS complexed with corilagin (3G′) or 1,2,3,4,6-O-pentagalloyl glucose (5G). Values with different letters represent significant difference at *p* < 0.05.

SEC was used to investigate the effects of complexation with corilagin and 1,2,3,4,6-O-pentagalloyl glucose on the chain-length distribution of debranched HAMS, and the results are shown in Figure 1. All distributions were normalized to the same peak maximum to minimize the influence of differences in sample concentration. A typical chain-length distribution was observed for debranched HAMS, consisting of three characteristic peaks. The AP1 region (X < 32) and AP2 region (32 < X < 300) correspond to shorter amylopectin chains and longer amylopectin branches, respectively. The AM peak (X, 300~100,000) corresponds to amylose chains. Based on the area ratio of the amylose content region to the whole chain-length distribution, the amylose content of HAMS was calculated to be 59.03%.

After treatment without polyphenols, the chain-length distribution of amylopectin showed no obvious change compared with that of HAMS, although slight variations were observed. In contrast, the AM peak increased markedly, with the amylose content increased from 59.03% in HAMS to 65.42% after treatment without polyphenols. Upon complexation with corilagin and 1,2,3,4,6-O-pentagalloyl glucose, the AM peak further increased, and the amylose content reached 73.67% and 76.56%, respectively, indicating that treatment with the two polyphenols increased the relative amylose content in the resulting complexes. This result agrees with a previous study showing that complexation with gallic acid increased the relative amylose content in starch complexes, possibly because gallic acid preferentially interacted with amylose, and the resulting complexes were insoluble and therefore more readily recovered during the preparation process, while part of the uncomplexed or more soluble amylopectin fractions may have been lost during washing [24]. This phenomenon is more apparent for 1,2,3,4,6-O-pentagalloyl glucose than corilagin, possibly because differences in the complexation of the two polyphenols with high-amylose maize starch led to 1,2,3,4,6-O-pentagalloyl glucose–starch complexes being more readily collected during the preparation process.

### 3.2. Short-Range Ordered Structure

**Figure 2:**
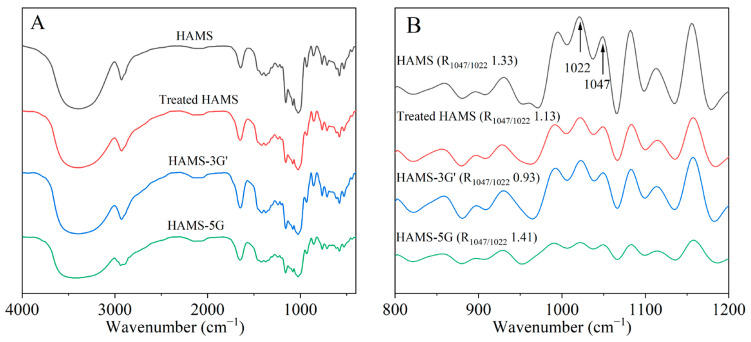
Short-range ordered structure of of high-amylose maize starch (HAMS), treated HAMS, HAMS complexed with corilagin (3G′) or 1,2,3,4,6-O-pentagalloyl glucose (5G), including (**A**) FTIR spectra and (**B**) deconvoluted FTIR spectra with R_1047/1022_ value.

Figure 2A shows the FTIR spectra of HAMS, treated HAMS, and HAMS complexed with corilagin or 1,2,3,4,6-O-pentagalloyl glucose in the range of 4000~400 cm^−1^. For starch samples, the absorption peak around 3390 cm^−1^ corresponds to the O–H stretching vibration, while the peak at 2925 cm^−1^ is attributed to the symmetric stretching vibration of CH_2_. The range from 1200 to 800 cm^−1^ is regarded as the fingerprint region of starch [28,29]. Compared with HAMS, no obvious shift in the absorption peaks was observed in the fingerprint region of treated HAMS, suggesting that the treatment did not cause obvious changes in the characteristic chemical groups of starch. Similarly, no new absorption peaks were detected in the spectra of the HAMS-corilagin and HAMS-1,2,3,4,6-O-pentagalloyl glucose complexes, although minor changes in peak intensity were observed. This observation is consistent with previous studies on the FTIR spectra of starch complexed with longan seed polyphenols and lotus leaf flavonoids [20,30]. These results indicate that there are no new covalent bonds formed in HAMS-corilagin and HAMS-1,2,3,4,6-O-pentagalloyl glucose complexes, and that the interactions between HAMS and the two polyphenols were mainly driven by non-covalent forces.

To further evaluate the short-range ordered structure of starch samples, the spectra in the fingerprint region (800~1200 cm^−1^) were deconvoluted, as shown in Figure 2B. The bands at 1047 and 1022 cm^−1^, corresponding to ordered and amorphous structures, respectively, were then analyzed [31,32]. The intensity ratio of the bands at 1047 and 1022 cm^−1^ (labelled as R_1047/1022_) was calculated to reflect the short-range ordered structure of starch samples, and the corresponding values are shown in Figure 2B. The R_1047/1022_ value of HAMS was 1.33 and decreased to 1.13 after being treated without polyphenols, which is consistent with previous reports showing that thermal treatment leads to a loss of the short-range ordered structure of starch [33]. Complexation with corilagin led to a further decrease in the R_1047_/_1022_ value to 0.93, while complexation with 1,2,3,4,6-O-pentagalloyl glucose increased the value to 1.41. These results indicate that the two polyphenols exhibited different effects on the short-range ordered structure of the starch complex, suggesting possible differences in non-covalent interactions with high-amylose maize starch.

### 3.3. Crystalline Structrue

**Figure 3:**
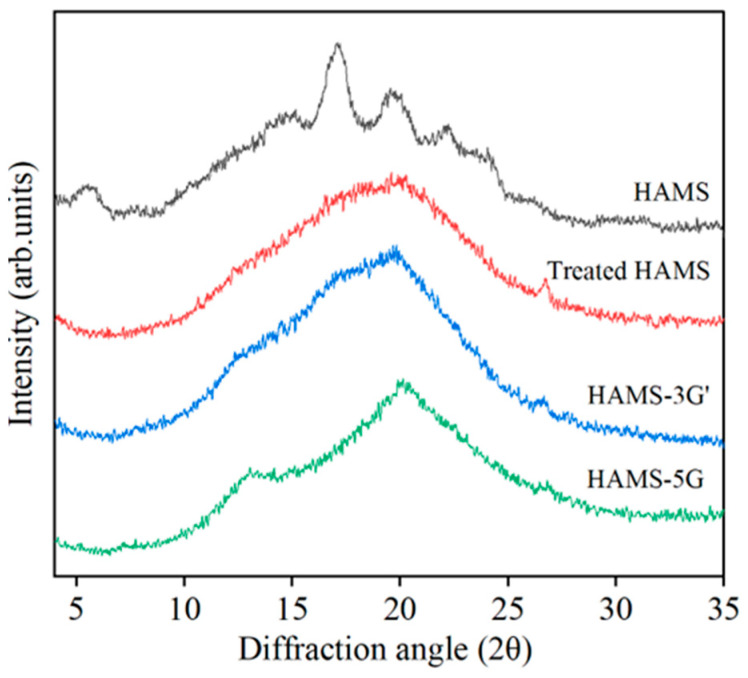
X-ray diffraction patterns of high-amylose maize starch (HAMS), treated HAMS, and HAMS complexed with corilagin (3G’) or 1,2,3,4,6-O-pentagalloyl glucose (5G).

XRD was used to investigate the differences in the crystalline structure of HAMS after treatment with corilagin or 1,2,3,4,6-O-pentagalloyl glucose. Figure 3 exhibits the XRD patterns of HAMS, treated HAMS, and the HAMS complexes prepared with corilagin and 1,2,3,4,6-O-pentagalloyl glucose. HAMS showed diffraction peaks at 5.4°, 16.3°, 20.3° and 22.9° (2θ), indicating a typical B-type crystalline structure. After HAMS was treated without polyphenols (denoted by treated HAMS in Figure 3), the diffraction peaks of HAMS almost disappeared, suggesting that the native crystalline structure of HAMS was largely disrupted during the treatment process [34]. No obvious diffraction peaks were observed for HAMS treated with corilagin, suggesting that corilagin did not promote the formation of a detectable crystalline structure. Conversely, HAMS treated with 1,2,3,4,6-O-pentagalloyl glucose exhibited new diffraction peaks at 7.2°, 13.0°, and 20.0° (2θ), which have been reported as typical peaks of a V-type crystalline structure [35,36]. These suggest that 1,2,3,4,6-O-pentagalloyl glucose can form V-type crystals with HAMS. These differences in the crystalline structure of the corilagin-HAMS and 1,2,3,4,6-O-pentagalloyl glucose-HAMS complexes were consistent with the differences in the short-range ordered structure of the two complexes (Figure 2). Combined with the above results, these differences are likely attributed to the different interaction types of the two polyphenols with high-amylose maize starch.

### 3.4. Isothermal Titration Calorimetry

**Figure 4:**
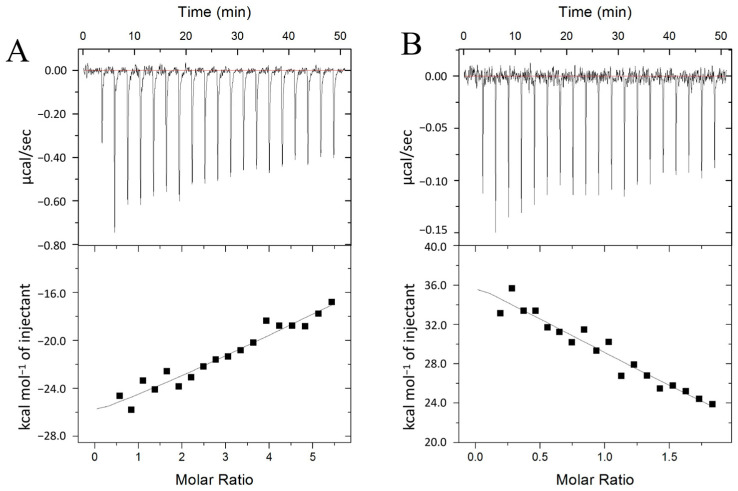
Raw thermograms (**upper panels**) and integrated binding isotherms (**lower panels**) for the titration of corilagin (**A**) and 1,2,3,4,6-O-pentagalloyl glucose (**B**) into high-amylose maize starch.

Isothermal titration calorimetry was used to compare the thermodynamics parameters of the interactions between HAMS and corilagin or 1,2,3,4,6-O-pentagalloyl glucose. The raw thermograms and integrated binding isotherms obtained from the titration of corilagin and 1,2,3,4,6-O-pentagalloyl glucose into high-amylose maize starch are presented in Figure 4A and 4B, respectively. The upper panels show the raw ITC thermograms for the interactions between the polyphenols and high-amylose maize starch, whereas the lower panels present the corrected integrated heats for each injection. As shown in the lower panels, corilagin and 1,2,3,4,6-O-pentagalloyl glucose exhibited different binding isotherms toward high-amylose maize starch, indicating differences in the binding behavior of the two polyphenols toward the high-amylose maize starch. To better compare the differences in binding between corilagin and 1,2,3,4,6-O-pentagalloyl glucose toward high-amylose maize starch, the thermodynamic parameters obtained from ITC fitting, including binding stoichiometry (*n*), equilibrium binding constants (Ka), enthalpy change (ΔH), entropy change (ΔS), and Gibbs free energy (ΔG = ΔH − TΔS), are summarized in Table 1.

As shown in Table 1, the Ka values for the binding of corilagin and 1,2,3,4,6-O-pentagalloyl glucose to HAMS were 5.17 × 10^4^ M^−1^ and 8.23 × 10^4^ M^−1^, respectively, indicating that both polyphenols were able to bind to HAMS. In addition, the Ka value of 1,2,3,4,6-O-pentagalloyl glucose was higher than the Ka value of corilagin, indicating that 1,2,3,4,6-O-pentagalloyl glucose had a stronger binding affinity for HAMS than corilagin. In contrast, the binding stoichiometry (*n*), representing the number of binding sites, was 10.40 for corilagin binding to HAMS and 4.06 for 1,2,3,4,6-O-pentagalloyl glucose binding to HAMS. This indicates that corilagin bound to HAMS at a larger number of sites than 1,2,3,4,6-O-pentagalloyl glucose. Combined with the Ka values, these results might suggest that corilagin bound to HAMS at more sites but with lower affinity, whereas 1,2,3,4,6-O-pentagalloyl glucose bound to HAMS at fewer sites but with stronger affinity [37]. In terms of enthalpy change (ΔH), the value for the binding of corilagin to HAMS was negative (−3.39 × 10^4^ cal·mol^−1^), suggesting that the binding of corilagin to HAMS was an exothermic process. However, the ΔH value for the binding of 1,2,3,4,6-O-pentagalloyl glucose to HAMS was positive (5.39 × 10^4^ cal·mol^−1^), indicating that the binding of 1,2,3,4,6-O-pentagalloyl glucose to HAMS was an endothermic process. The Gibbs free energy change (ΔG) values for the binding of corilagin and 1,2,3,4,6-O-pentagalloyl glucose to HAMS were −6.98 × 10^3^ and −7.20 × 10^3^ cal·mol^−1^, respectively, indicating that the binding of both polyphenols to HAMS occurred spontaneously. For corilagin, the negative ΔS value (−83.40 cal·K^−1^·mol^−1^), together with the negative ΔH value, indicates that the binding of corilagin to HAMS was mainly through hydrogen bonding and/or van der Waals interaction [20,37]. In contrast, for 1,2,3,4,6-O-pentagalloyl glucose, the positive ΔS value (189 cal·K^−1^·mol^−1^), together with the positive ΔH value, were consistent with a binding process involving a substantial contribution from hydrophobic interactions [20,38]. These thermodynamic differences may further reflect the influence of galloyl accessibility on the interaction patterns of the two polyphenols with HAMS. Specifically, relatively accessible galloyl groups may favor contact with starch hydroxyl groups and contribute to hydrogen bonding, whereas less accessible or more sterically constrained galloyl groups may reduce hydrogen-bonding opportunities and make hydrophobic association more important. These results suggested that corilagin and 1,2,3,4,6-O-pentagalloyl glucose interacted with HAMS through different interaction types, which supported the findings from the SEC, FTIR, and XRD analyses.

### 3.5. Molecular Dynamics Simulation

**Figure 5:**
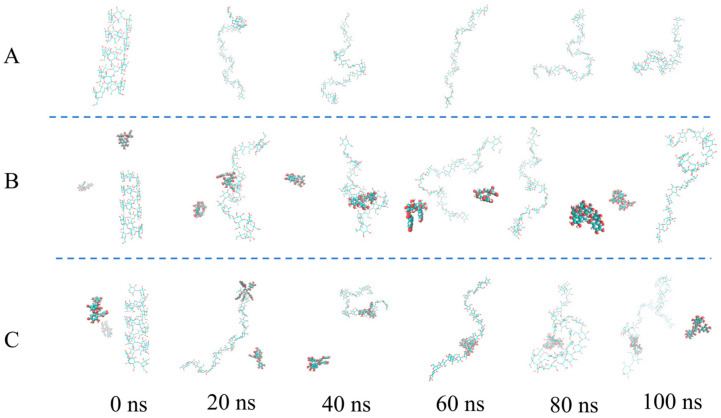
Snapshots of the spatial conformations of amylose alone (**A**), the complex of corilagin (3G’) with amylose (**B**), and the complex of 1,2,3,4,6-O-pentagalloyl glucose (5G) with amylose (**C**) during the 100 ns molecular dynamics simulation.

Molecular dynamics (MD) simulations were performed to further elucidate and visualize the molecular interactions between corilagin and high-amylose maize starch, as well as between 1,2,3,4,6-O-pentagalloyl glucose and high-amylose maize starch. A simplified amylose chain was employed as a representative model of high-amylose maize starch to simulate its interactions with the two polyphenols at the molecular level. Figure 5 shows the conformational evolution of single-helical amylose (Figure 5A), amylose–corilagin (Figure 5B), and amylose–1,2,3,4,6-O-pentagalloyl glucose (Figure 5C) over 100 ns of simulation. The control single-helical amylose showed irregular folding and continuous structural variation during the simulation, consistent with the previous reports [39,40]. In the corilagin–amylose system, corilagin gradually approached the amylose segment during the first 40 ns, but remained separated from the amylose segment after 40 ns. For the 1,2,3,4,6-O-pentagalloyl glucose and amylose system, at 20 ns, the ligand was bound to the amylose segment, and at 40 and 60 ns, the ligand was still bound to the amylose helix, although small changes were observed in the conformation. These results indicate that compared to corilagin, 1,2,3,4,6-O-pentagalloyl glucose exhibited a greater tendency to form and maintain a stable association with the amylose helix during the simulation.

**Figure 6:**
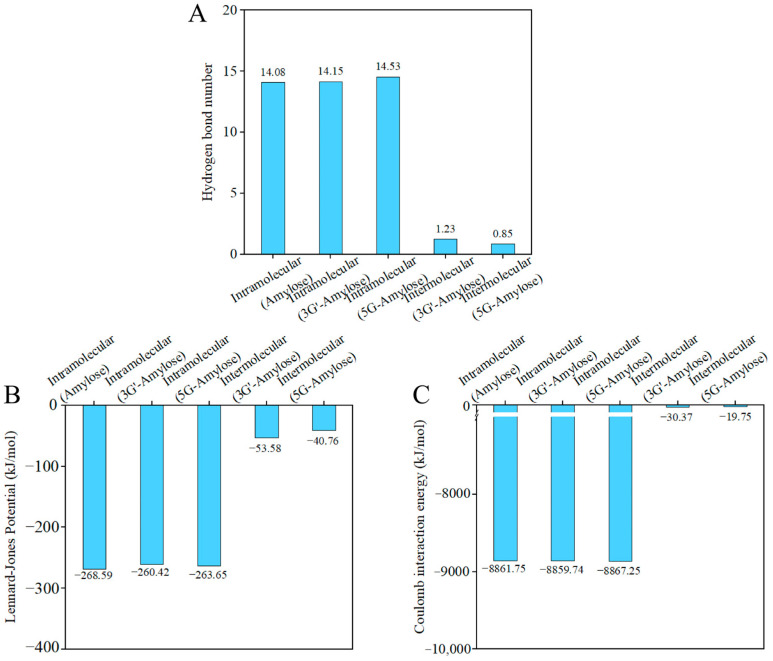
Non-covalent interaction forces in the intramolecular amylose system and in the intra- and intermolecular corilagin (3G′)–amylose and 1,2,3,4,6-O-pentagalloyl glucose (5G)–amylose systems, including the number of Hydrogen bonds (**A**), Lennard-Jones potential (**B**), and Coulomb interaction energy (**C**).

To further elucidate the non-covalent interactions in the amylose system and in the corilagin–amylose and 1,2,3,4,6-O-pentagalloyl glucose–amylose systems, the number of hydrogen bonds, Lennard-Jones potential, representing van der Waals interactions, and Coulomb interaction energy, representing electrostatic interactions were calculated, with results shown in Figure 6. Figure 6A shows that the number of intramolecular hydrogen bonds in the amylose-only system was 14.08, which was similar to that in the corilagin–amylose system (14.15), but lower than that in the 1,2,3,4,6-O-pentagalloyl glucose–amylose system (14.53). In addition, the intramolecular Lennard-Jones potential and Coulomb interaction energy of the amylose-only system were similar to those of the corilagin–amylose and 1,2,3,4,6-O-pentagalloyl glucose–amylose systems (Figure 6B,C). These results suggest that corilagin exerted limited influence on amylose structure, whereas 1,2,3,4,6-O-pentagalloyl glucose showed a slight tendency to stabilize the helical structure of amylose, which further supported the above XRD results (shown in Figure 3). For the intermolecular interactions, the corilagin–amylose system exhibited slightly higher values than the 1,2,3,4,6-O-pentagalloyl glucose–amylose system in terms of the number of hydrogen bonds, as well as the absolute values of the intermolecular Lennard-Jones potential and Coulomb interaction energy. These results indicate that, compared with the corilagin–amylose system, the 1,2,3,4,6-O-pentagalloyl glucose–amylose system had relatively lower contributions from hydrogen bonding, van der Waals interactions, and electrostatic interactions. Combined with the ITC results, this further suggests that hydrophobic interaction is likely the main force for the formation of a 1,2,3,4,6-O-pentagalloyl glucose–HAMS complex, whereas hydrogen bonding and van der Waals interactions are mainly involved in the formation of the corilagin–HAMS complex. It should be noted that the molecular dynamics simulations were performed using a simplified amylose model. This model cannot fully represent the structural complexity of real HAMS systems, which contain amylose, amylopectin, crystalline and amorphous regions, and heterogeneous chain conformations. Therefore, the simulation results should be interpreted as molecular-level support for possible amylose–polyphenol interactions rather than as a complete representation of the whole starch system.

## 4. Conclusions

To support the valorization of antioxidant polyphenols from fruit and vegetable processing by-products, this study compared the interactions of corilagin and 1,2,3,4,6-O-pentagalloyl glucose with high-amylose maize starch (HAMS), focusing on how the number and accessibility of galloyl moieties affect starch–polyphenol complexation mechanisms.

SEC results showed that the amylopectin chain-length distributions of the two HAMS–polyphenol complexes were similar to that of treated HAMS, whereas their relative amylose contents increased, indicating that both polyphenols preferentially interacted with amylose. The higher relative amylose content observed in the HAMS–corilagin complex compared to the HAMS–1,2,3,4,6-O-pentagalloyl glucose complex may be related to differences in complex formation and recovery during sample preparation. FTIR analysis showed that no new covalent bonds were formed, indicating that both complexes were mainly stabilized by non-covalent interactions. However, XRD and short-range order analyses revealed distinct structural effects. Corilagin reduced the short-range ordered structure of HAMS and did not induce detectable crystalline structure, whereas 1,2,3,4,6-O-pentagalloyl glucose increased short-range order and promoted the formation of a V-type crystalline structure. ITC analysis further showed that corilagin had more binding sites but lower binding affinity toward HAMS than 1,2,3,4,6-O-pentagalloyl glucose. The negative ΔH and ΔS values for corilagin suggested that its interaction with HAMS was mainly mediated by hydrogen bonding and/or van der Waals interactions, whereas the positive ΔH and ΔS values for 1,2,3,4,6-O-pentagalloyl glucose indicated a hydrophobic interaction-dominated binding process. MD simulations supported these thermodynamic results. Compared with corilagin, 1,2,3,4,6-O-pentagalloyl glucose showed a greater tendency to form and maintain a stable association with the amylose helix. Moreover, the relatively lower contributions of hydrogen bonding, van der Waals interactions, and electrostatic interactions in the 1,2,3,4,6-O-pentagalloyl glucose–amylose system further supported the dominant role of hydrophobic interactions in its complexation with HAMS.

Taken together, these findings suggest that the number and accessibility of galloyl moieties are important structural factors influencing the interaction mechanisms between galloyl-based polyphenols and HAMS. The non-covalent complexes formed in this study may exhibit relative stability through multiple cooperative interactions, which could influence polyphenol retention/release and starch digestibility during processing or digestion. These results provide guidance for the rational design of starch–polyphenol complexes and support the targeted utilization of antioxidant polyphenols from fruit and vegetable processing by-products in functional starch-based foods and nutraceutical delivery systems, although their effects on bioavailability, glycemic response, and digestion behavior require further validation.

## Figures and Tables

**Table 1:** Thermodynamic parameters for the respective binding of corilagin (3G’) and 1,2,3,4,6-O-pentagalloyl glucose (5G) to high-amylose maize starch (HAMS).

Ligand	Receptor	Ka (M^−1^)	*n* (Sites)	ΔH (cal·mol^−1^)	ΔS (cal·K^−1^·mol^−1^)	ΔG (cal·mol^−1^)
3G’	HAMS	5.17 × 10^4^	10.40	−3.39 × 10^4^	−83.40	−6.98 × 10^3^
5G	HAMS	8.23 ×10^4^	4.06	5.39 × 10^4^	189.00	−7.20 × 10^3^

## Data Availability

The original contributions presented in this study are included in the article. Further inquiries can be directed to the corresponding author(s).

## Abbreviations

3G’	Corilagin
5G	1,2,3,4,6-O-pentagalloyl glucose
HAMS	High-amylose maize starch
SEC	Size-exclusion chromatography
FTIR	Fourier transform infrared spectroscopy
XRD	X-ray diffraction
ITC	Isothermal titration calorimetry
MD	Molecular dynamics
DMSO	Dimethyl sulfoxide

## References

[1] Deng N., Deng Z., Tang C., Liu C., Luo S., Chen T., Hu X. (2021). Formation, structure and properties of the starch-polyphenol inclusion complex: A review. Trends Food Sci. Technol..

[2] Han B., Ma Q., Yan S., Jia S., Hu Y., Xu H., Liu S., Zhang H., Wang P., Guo J. (2026). Recent advances in the formation, characterization, and applications of starch-polyphenol complexes: A review. Food Chem..

[3] Salimi M., Channab B.E., El Idrissi A., Zahouily M., Motamedi E. (2023). A comprehensive review on starch: Structure, modification, and applications in slow/controlled-release fertilizers in agriculture. Carbohydr. Polym..

[4] Wang R., Li M., Brennan M.A., Dhital S., Kulasiri D., Brennan C.S., Guo B. (2023). Complexation of starch and phenolic compounds during food processing and impacts on the release of phenolic compounds. Crit. Rev. Food Sci. Nutr..

[5] He T., Zhang X., Zhao L., Zou J., Qiu R., Liu X., Hu Z., Wang K. (2023). Insoluble dietary fiber from wheat bran retards starch digestion by reducing the activity of alpha-amylase. Food Chem..

[6] He T., Zhao L., Chen Y., Zhang X., Hu Z., Wang K. (2021). Longan seed polyphenols inhibit alpha-amylase activity and reduce postprandial glycemic response in mice. Food Funct..

[7] Zagoskina N.V., Zubova M.Y., Nechaeva T.L., Kazantseva V.V., Goncharuk E.A., Katanskaya V.M., Baranova E.N., Aksenova M.A. (2023). Polyphenols in Plants: Structure, Biosynthesis, Abiotic Stress Regulation, and Practical Applications (Review). Int. J. Mol. Sci..

[8] Zheng F., Ren F., Zhu X., Han Z., Jia Y., Liu X., Chen B., Liu H. (2025). The interaction between starch and phenolic acids: Effects on starch physicochemical properties, digestibility and phenolic acids stability. Food Funct..

[9] Gutiérrez T.J., Tovar J. (2021). Update of the concept of type 5 resistant starch (RS5): Self-assembled starch V-type complexes. Trends Food Sci. Technol..

[10] Dong H., Ding Q., Jiang Y., Li X., Han W. (2021). Pickering emulsions stabilized by spherical cellulose nanocrystals. Carbohydr. Polym..

[11] Wu Y., Liu Y., Jia Y., Feng C.H., Ren F., Liu H. (2024). Research progress on the regulation of starch-polyphenol interactions in food processing. Int. J. Biol. Macromol..

[12] Luo D., Fan J., Jin M., Zhang X., Wang J., Rao H., Xue W. (2024). The influence mechanism of pH and polyphenol structures on the formation, structure, and digestibility of pea starch-polyphenol complexes via high-pressure homogenization. Food Res. Int..

[13] Zuo Y., Zhu F., Jiang S., Sui Z., Kong X. (2024). Differences in structure, physicochemical properties, and in vitro digestibility of three types of starch complexed with tannic acid. Food Hydrocoll..

[14] Fan H., Yao X., Chen Z., Ma R., Wen Y., Li H., Wang J., Sun B. (2024). Interaction of high amylose corn starch with polyphenols: Modulating the stability of polyphenols with different structure against thermal processing. Food Chem..

[15] Liu L., Jia W., Jiang S., Zhang G., Zhao J., Xu J., Wang L., Wu D., Tao J., Yue H. (2023). Inhibitory activities and rules of plant gallotannins with different numbers of galloyl moieties on sucrase, maltase and alpha-amylase in vitro and in vivo. Phytomedicine.

[16] Zheng Y., Chen T., Li K., Wang L., Liu X., Zhao L., Hu Z., Wang K. (2025). V-type starch-galloyl-based polyphenol complex stimulates GLP-1 and PYY secretion and improves satiety in mice. Carbohydr. Polym..

[17] Shahidi F., Athiyappan K.D. (2025). Polyphenol-polysaccharide interactions: Molecular mechanisms and potential applications in food systems—A comprehensive review. Food Prod. Process Nutr..

[18] Cao J., Yan S., Xiao Y., Han L., Sun L., Wang M. (2022). Number of galloyl moiety and intramolecular bonds in galloyl-based polyphenols affect their interaction with alpha-glucosidase. Food Chem..

[19] Cao J., Zhang Y., Han L., Zhang S., Duan X., Sun L., Wang M. (2020). Number of galloyl moieties and molecular flexibility are both important in alpha-amylase inhibition by galloyl-based polyphenols. Food Funct..

[20] He T., Wang K., Zhao L., Chen Y., Zhou W., Liu F., Hu Z. (2021). Interaction with longan seed polyphenols affects the structure and digestion properties of maize starch. Carbohydr. Polym..

[21] Wang K., Hasjim J., Wu A.C., Li E., Henry R.J., Gilbert R.G. (2015). Roles of GBSSI and SSIIa in determining amylose fine structure. Carbohydr. Polym..

[22] Cave R.A., Seabrook S.A., Gidley M.J., Gilbert R.G. (2009). Characterization of starch by size-exclusion chromatography: The limitations imposed by shear scission. Biomacromolecules.

[23] Lai K., He P., Van der Meeren P., Zhang B., Fu X., Gao Q., Huang Q. (2026). Volatility-tuned encapsulation in V-type starch: Phase-dependent efficiency and release kinetics. Food Hydrocoll..

[24] He T., Zhao L., Wang L., Liu L., Liu X., Dhital S., Hu Z., Wang K. (2024). Gallic acid forms V-amylose complex structure with starch through hydrophobic interaction. Int. J. Biol. Macromol..

[25] Abraham M.J., Murtola T., Schulz R., Páll S., Smith J.C., Hess B., Lindahl E. (2015). GROMACS: High performance molecular simulations through multi-level parallelism from laptops to supercomputers. SoftwareX.

[26] Huang J., MacKerell A.D. (2013). CHARMM36 all-atom additive protein force field: Validation based on comparison to NMR data. J. Comput. Chem..

[27] Gao Q., Sun Y., He R., Zheng J., Zhang B., Tan C.P., Fu X., Huang Q. (2023). Molecular encapsulation of cinnamaldehyde in V-type starch: The role of solvent and temperature. Food Hydrocoll..

[28] Althawab S.A., Amoako D.B., Annor G.A., Awika J.M. (2023). Stability of starch-proanthocyanidin complexes to in-vitro amylase digestion after hydrothermal processing. Food Chem..

[29] Chen N., Feng Z.J., Gao H.X., He Q., Zeng W.C. (2024). Elucidating the influence and mechanism of different phenols on the properties, food quality and function of maize starch. Food Chem..

[30] Wang M., Chen J., Chen S., Ye X., Liu D. (2021). Inhibition effect of three common proanthocyanidins from grape seeds, peanut skins and pine barks on maize starch retrogradation. Carbohydr. Polym..

[31] Ma Y., Chen Z., Wang Z., Chen R., Zhang S. (2023). Molecular interactions between apigenin and starch with different amylose/amylopectin ratios revealed by X-ray diffraction, FT-IR and solid-state NMR. Carbohydr. Polym..

[32] Lu H., Tian Y., Ma R. (2023). Assessment of order of helical structures of retrograded starch by Raman spectroscopy. Food Hydrocoll..

[33] Liu X., Huang S., Chao C., Yu J., Copeland L., Wang S. (2022). Changes of starch during thermal processing of foods: Current status and future directions. Trends Food Sci. Technol..

[34] Gonzalez A., Wang Y.-J. (2023). Effects of acid hydrolysis level prior to heat-moisture treatment on properties of starches with different crystalline polymorphs. Lwt-Food Sci. Technol..

[35] Tan L., Kong L. (2020). Starch-guest inclusion complexes: Formation, structure, and enzymatic digestion. Crit. Rev. Food Sci. Nutr..

[36] Deng X., Ng W.H., Zhou Z., Li C. (2026). Current understanding of starch-polyphenol complexes and their potential in vivo metabolism, impact on gut microbiota and treating diabetes. Crit. Rev. Food Sci. Nutr..

[37] Yu M., Liu B., Zhong F., Wan Q., Zhu S., Huang D., Li Y. (2021). Interactions between caffeic acid and corn starch with varying amylose content and their effects on starch digestion. Food Hydrocoll..

[38] Ross P.D., Subramanian S. (1981). Thermodynamics of protein association reactions: Forces contributing to stability. Biochemistry.

[39] Gao Q., Bie P., Tong X., Zhang B., Fu X., Huang Q. (2021). Complexation between High-Amylose Starch and Binary Aroma Compounds of Decanal and Thymol: Cooperativity or Competition?. J. Agric. Food Chem..

[40] Lopez C.A., de Vries A.H., Marrink S.J. (2012). Amylose folding under the influence of lipids. Carbohydr. Res..

